# Ineffectual immunity in a resurrected mouse model of persistent viremia

**DOI:** 10.1128/jvi.00248-25

**Published:** 2025-05-08

**Authors:** Kylie Nennig, Teressa Shaw, Logan Borsinger, Adam L. Bailey

**Affiliations:** 1 Department of Pathology and Laboratory Medicine, University of Wisconsin–Madison School of Medicine and Public Health 189586, Madison, Wisconsin, USA; New York University Department of Microbiology, New York, New York, USA

**Keywords:** viral persistence, chronic infection, viremia, arterivirus, lymphocytic choriomeningitis virus, LCMV, lactate dehydrogenase elevating virus, LDV, failed immunity, immune tolerance, immune failure

## Abstract

Viruses that establish persistent (i.e., chronic) infections have evolved sophisticated strategies to avoid clearance by the host immune system. This is particularly true for viruses that infect immunocompetent mammals and sustain high infectious burdens in body sites under intense immune surveillance (i.e., the blood, a.k.a., “viremia”). Historically, lymphocytic choriomeningitis virus (LCMV) infection of laboratory mice has served as a powerful model to understand mechanisms of failed immunity, but other viruses may have unique and underappreciated persistence strategies. Here, we resurrect a bygone model of viral persistence—lactate dehydrogenase-elevating virus (LDV)—and use modern transgenic mouse technologies to investigate various aspects of anti-viral immunity. We find that interferons have a modest impact on LDV replication, with interferon-alpha blunting LDV viremia in the acute phase of the infection and interferon-gamma reducing LDV viral loads in the chronic phase of infection, but only when paired with an intact interferon-alpha response. Adaptive immunity, assessed in Rag-knockout mice, had only a modest impact on LDV viremia, and only during the sub-acute phase of infection. Mice lacking the critical immune checkpoint molecule PD-1 showed no signs of disease and supported LDV viral loads at levels equivalent to their wild-type counterparts. Altogether, these results point to a novel and highly effective mechanism of persistence that is minimally impacted by conventional aspects of anti-viral immunity or immune exhaustion—a rarity among persistent viruses. Given the relative paucity of chronic infection models in the laboratory mouse, LDV infection may be useful for exploring unique modes of immune system failure.

**IMPORTANCE:**

Viruses that infect a host over long periods of time have evolved unique strategies to evade the host immune system. Of particular interest are viruses that cause persistent infection in the laboratory mouse—the most well-developed tool for studying the mammalian immune system. Here, we resurrected a model of persistent RNA virus infection (lactate dehydrogenase-elevating virus, LDV) and applied modern tools of mouse immunology to further characterize its persistence. We found that host factors that typically have a dramatic effect on viral infections—e.g., the interferon system and lymphocytes—had very little impact on LDV infection. Removing “checks” on immune activation also had little effect on the virus or host health. Altogether, these findings imply that LDV uses a unique and highly effective mechanism to avoid immune clearance. Understanding this mechanism has implications for understanding ways in which the immune system fails.

## INTRODUCTION

Viruses capable of persistently infecting an immunocompetent host have developed sophisticated strategies for evading the host immune response. Common themes among viral persistence strategies include infection of immune-privileged sites (e.g., the central nervous system, CNS), latency, and the accumulation of mutations in key immune-targeting epitopes (i.e., immune escape) (1, 2). In mammals, the combination of innate and adaptive immune responses is so effective that viruses must often employ several of these strategies in combination in order to persist. For example, human immunodeficiency virus (HIV) rapidly accumulates mutations to escape antibody and T-cell responses while also establishing latent reservoirs in infected cells, some of which hide in the CNS; herpes simplex viruses (i.e., HSV-1 and HSV-2) enter latency in neurons of the CNS, where they can lie dormant for decades before reactivating (3). Although the immune evasion strategies used by persistent viruses may seem similar, the molecular mechanisms employed in their execution have no evolutionary relation—an example of convergent evolution.

Understanding the mechanism(s) by which a virus can persistently infect an immunocompetent host can elucidate novel mechanisms of “failed immunity,” revealing fundamental principles of the immune system in the process. For this, arguably no model has been more influential than lymphocytic choriomeningitis virus (LCMV), which can establish semi-persistent viremia in mice infected during the fetal/neonatal period (4). Leveraging the vast array of tools available for studying the laboratory mouse, investigations of LCMV persistence over the past 80 years have included the first examples of T-cell-mediated lysis of infected cells (5), major histocompatibility complex restriction of T-cell recognition (6), antiviral effect of immune cell adoptive transfer (7), and substantial contributions toward identifying and understanding the immune phenomena of exhaustion, tolerance, and checkpoint blockade (8, 9). These discoveries have spawned entirely new fields of research and found technological and biomedical applications far beyond their initial context. Yet, despite the fundamental discoveries made using LCMV, contemporary research on persistent viral infections has increasingly focused on viruses that cause disease in humans, namely HIV, hepatitis C virus, and hepatitis B virus. Importantly, these viruses do not infect laboratory mice, leaving this host—and the highly sophisticated tools that come with it—underutilized in the study of viral persistence.

Viruses in the *Arteriviridae* family (i.e., arteriviruses) are enveloped positive-sense single-stranded (+ss)RNA viruses that are distantly related to Coronaviruses (both are in the Order *Nidovirales*). The diversity of arteriviruses is vast, with a variety of mammals serving as natural hosts (10). Arterivirus infection can cause a variety of diseases, ranging from encephalitis, viral hemorrhagic fever, pneumonia, and spontaneous abortion. However, many arteriviruses persist as clinically silent, life-long infections in their natural host. Lactate dehydrogenase-elevating virus (LDV), the mouse arterivirus, was first described in 1960 as a “transmissible enzymic lesion” that was identifiable by the telltale elevation in serum lactate dehydrogenase (LDH) concentration in infected mice (11). LDV causes persistent high-titer viremia for the lifespan of the mouse without causing overt disease; however, research in the 1960–1980s demonstrated a number of significant impacts on the murine immune system (12, 13). For example, acute LDV infection acts as an adjuvant for other non-viral antigens, while chronic LDV infection suppresses primary humoral and cellular immune responses (14, 15). LDV also markedly reduces the clearance capacity of the reticuloendothelial system, which contributes to elevated LDH concentrations via reduced clearance of circulating LDH (16, 17). Despite this foundational work, LDV fell into relative obscurity in the 1990s as LCMV became the dominant model for viral immunology in mice. As a consequence, modern research tools and techniques have not been applied to LDV, and we still do not understand how LDV (or arteriviruses in general) maintain persistence.

## RESULTS

### LDV persistently infects wild-type C57BL/6J mice

**Fig 1:**
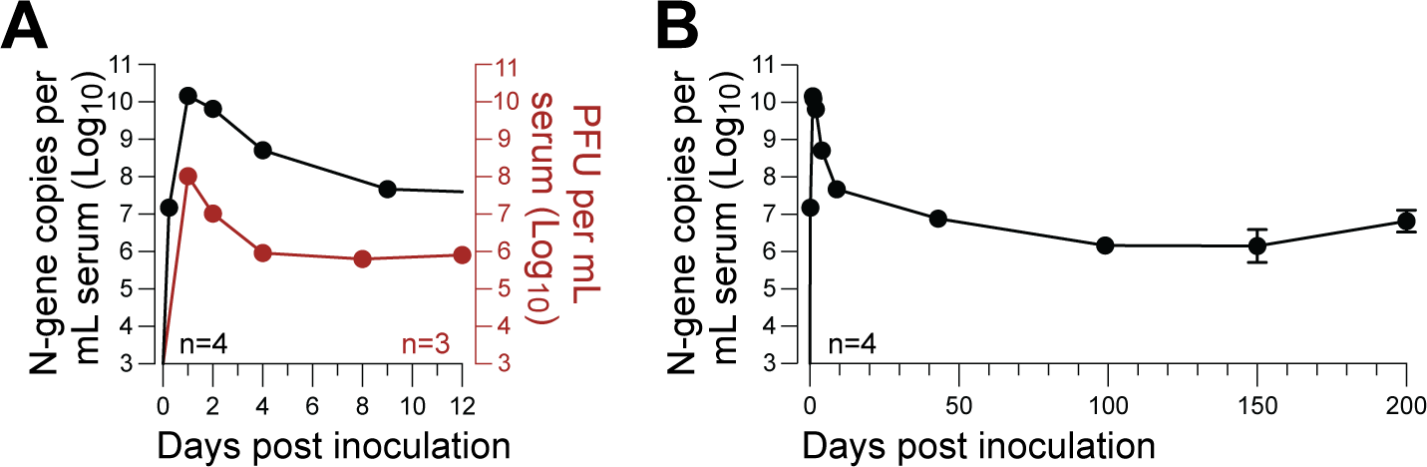
LDV persistently infects wild-type C57BL/6J mice. (**A**) Acute phase of LDV infection following intraperitoneal infection, with serum viral loads assessed by LDV-specific RT-qPCR (black, *n* = 4) or plaque assay (red, *n* = 3); error bars show ±SEM are too small to be shown. (**B**) Same data as panel A, with serum viral loads shown over 200 days assessed by LDV-specific RT-qPCR (black, *n* = 4).

To confirm prior literature, we first characterized LDV infection kinetics in adult C57BL/6J mice using a custom-built highly sensitive RT-qPCR assay specific for the LDV N-gene. High-resolution sampling during the first 48 hours of infection confirmed that peak viremia occurred within 24 hours post-inoculation, reaching a peak of >1 × 10^10^ N-gene copies per milliliter of serum in all animals (Fig. 1A). Viral loads rapidly declined over the following 10 days, followed by a further, more gradual decline until a stable set-point of ~1 × 10^7^ N-gene copies per milliliter of serum was established around 50–100 days post-inoculation (dpi) and maintained for the remainder of the study (Fig. 1B).

### Immune gene knockout has a minimal impact on LDV viremia

**Fig 2:**
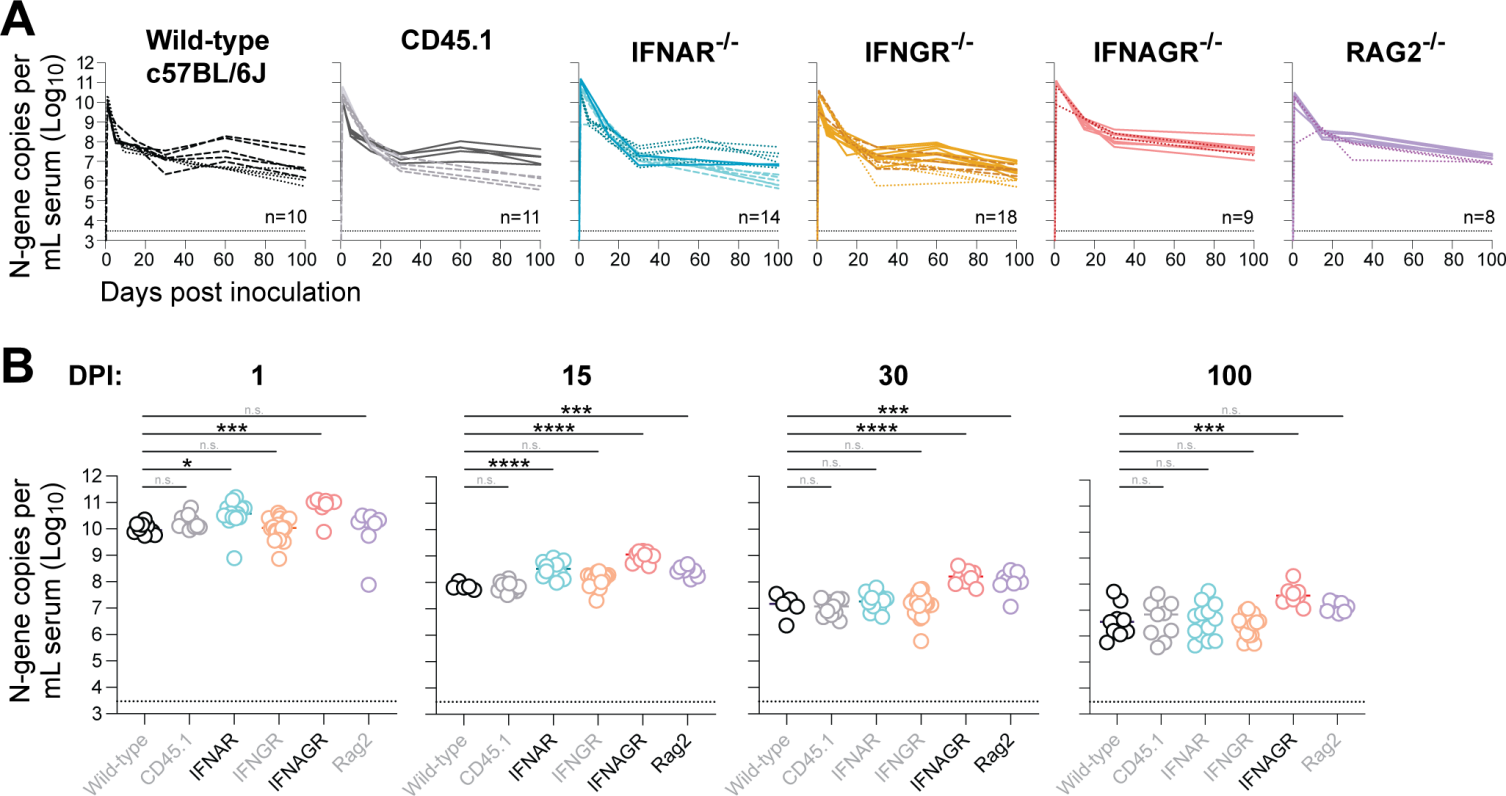
Immune gene knockout has minimal impact on LDV viremia. (**A**) Serum LDV viral loads in wild-type mice (black, *n* = 10), CD45.1 congenic mice (gray, *n* = 11), interferon-alpha receptor-deficient mice (blue, *n* = 14), interferon-gamma receptor-deficient mice (yellow, *n* = 8), interferon-alpha and -gamma receptor-deficient mice (red, *n* = 9), and Rag2-deficient mice (purple, *n* = 8) following intraperitoneal infection. Infection studies were performed in multiple independent cohorts, with each cohort represented by a unique line type (e.g., solid vs dashed). Thin dashed line indicates the limit of detection. (**B**) Same data as panel A, with comparisons to the wild-type group by one-way ANOVA using Dunnett’s test for multiple comparisons: ****P < 0.0001; ****P < 0.001; **P < 0.01; and *P < 0.05.

The innate and adaptive immune systems each play an important role in controlling and ultimately eradicating viral infections. Although LDV is clearly capable of avoiding eradication by the host immune system, we hypothesized that both the innate and adaptive immune systems would exert some degree of control over LDV infection, as has been shown for virtually all persistent viral infections to date. Leveraging the murine host, we infected mice genetically deficient in various genes critical for immunity and used RT-qPCR on RNA extracted from serum to assess body-wide LDV replication in infected individuals over time (Fig. 2). Mice lacking the interferon-alpha receptor (*IFNAR*^-/-^), which serves as a critical early mediator of the innate immune response, supported statistically higher LDV viral loads during the acute phase of infection (1 and 15 dpi) compared to the wild-type mice; however, the magnitude of this effect was small (3.5-fold at 1 dpi and 4.5-fold at 15 dpi) and became non-significant in the chronic phase (30 and 100 dpi). Single knockout of the pleiotropic cytokine interferon-gamma (*IFNGR*^-/-^) had no effect on LDV viral load at any point during infection, but double knockout of interferon-alpha and -gamma (*IFNAGR*^-/-^) resulted in higher viral loads in the chronic phase. Finally, we examined the impact of the adaptive lymphocyte response to partially control LDV replication by infecting mice that lack mature T and B cells (*RAG2*^-/-^). Although these mice supported slightly higher levels of LDV viremia in the sub-acute period (15 and 30 dpi), they sustained equivalent viral loads compared to their wild-type counterparts in chronic infection (100 dpi).

### PD-1 knockout has minimal impact on host health or LDV viremia

**Fig 3:**
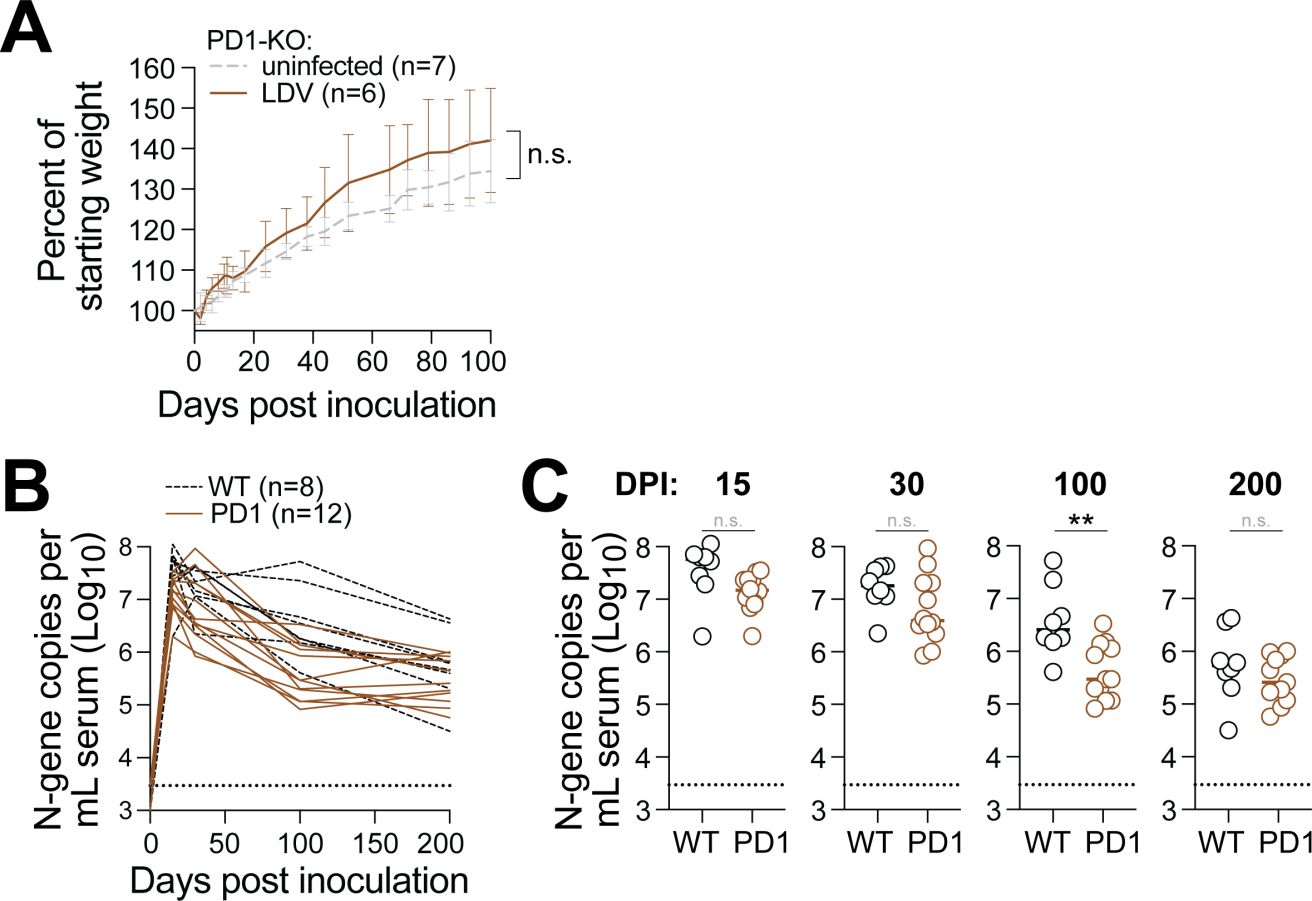
PD-1 knockout does not impact host health or LDV viremia**.** (**A**) Trajectory of weight gain in LDV-infected (brown, *n* = 6) vs uninfected (gray, *n* = 7) PD-1 knockout mice. Data were compiled from two independent experiments. Non-significance was determined by unpaired *t*-test at 100 dpi. (**B**) Trajectory of LDV viremia in wild-type (black, *n* = 8) vs PD-1 knockout mice (*n* = 12). (**C**) Same data as panel B, with statistical significance determined by unpaired *t*-test.

Given our finding that adaptive lymphocytes have little-to-no antiviral activity against chronic LDV infection, we wondered whether LDV-specific immune cells were becoming exhausted. Typically, this would be assessed by examining exhaustion markers on the surface of virus-specific lymphocytes; however, tools for labeling LDV-specific lymphocytes do not yet exist. To circumvent this limitation, we used mice that lack the master regulator of immune exhaustion: programmed cell death protein 1 (PD-1). Mice that lack PD-1 (or its ligand, PD-L1) have an over-exuberant immune response to foreign antigens that, if not left unchecked, will result in significant immunopathology (Fig. 3). For example, the persistent antigen stimulation that results from chronic LCMV infection leads to rapid weight loss and lethality in *PD-1*^-/-^ mice. Surprisingly, *PD-1*^-/-^ mice infected with LDV exhibited regular weight gain compared to their uninfected *PD-1*^-/-^ counterparts for 100 days after infection. To determine if *PD-1*^-/-^ mice were better able to control LDV replication, we monitored viremia for 200 days but found LDV viral loads to be equivalent between wild-type and *PD-1*^-/-^ mice, with the exception of 100 dpi, at which time LDV viremia was slightly lower in the *PD-1*^-/-^ mice compared to wild type.

## DISCUSSION

Viral persistence provides a unique window into immune system function (or dysfunction), with previously uncharacterized mechanisms of persistence illuminating potentially novel immune functions. Here, we show that LDV—a persistent +ssRNA virus that naturally infects mice—lacks many features of more well-characterized persistent viruses. Using a panel of mice with immune gene lesions, we show that neither the interferon response nor the adaptive immune system is able to exert substantial/sustained suppression of LDV replication. This suggests that LDV possesses a highly sophisticated arsenal of factors that facilitate immune suppression and evasion, as has been shown for the related arterivirus, porcine reproductive and respiratory syndrome virus (PRRSV), which infects pigs. Indeed, PRRSV possesses no less than six unique proteins capable of antagonizing the interferon pathway, with corresponding orthologs present in LDV. Antibody and T-cell responses to PRRSV are also notably weak, and mutational escape via the presentation of “decoy epitopes” also likely facilitates evasion of adaptive responses. However, much is still unknown regarding the molecular details of these evasion mechanisms, as their interrogation has been relatively limited by the porcine host.

Although our transgenic approach to interrogating immune control of LDV infection is by no means exhaustive, the overall lack of interferon- or lymphocyte-mediated control of viral replication implies that, unlike many other persistent viral infections, LDV viral load is determined almost entirely by target cell availability. LDV is thought to exclusively infect tissue-resident macrophages, and recent work by our lab has confirmed that the macrophage-specific hemoglobin-haptoglobin scavenger receptor CD163 is required for LDV infection *in vitro* and *in vivo*. Nevertheless, a more thorough delineation of the specific macrophage subsets that support LDV replication in each tissue at various stages of infection is needed to further understand several unique features of LDV infection that include immune antagonism, lack of immunopathology resulting from T cell exhaustion, and lack of discernible disease. Understanding these aspects of “non-pathogenic” LDV infection could also serve as a useful comparator against highly pathogenic arterivirus infections (e.g., PRRSV).

Ultimately, the “negative” data gleaned from this study seem sufficient to highlight the unique—albeit still poorly understood—mechanism(s) of LDV persistence. Given the success of other persistent viruses as immunology tools (e.g., LCMV, murine norovirus, and murine gamma herpesvirus), it seems that much could be gained from further study of LDV.

## MATERIALS AND METHODS

### Virus

Material containing the LDV Plagemann strain (GenBank accession OQ570966) was generously provided by Kay Faaberg (USDA Ames). An LDV stock was generated by inoculating a C57BL/6J laboratory mouse with ≈50 μL of source material and euthanized via CO_2_ asphyxiation at 24 hours post-inoculation, at which time a maximal blood draw was performed. Purified sera were then diluted in phosphate-buffered saline + 2% heat-inactivated fetal calf serum (Omega Scientific, Tarzana, CA, USA) and frozen in 300-μL aliquots at −80°C. The stock was determined to contain 1.42 × 10^8^ N gene copies per milliliter and 6 × 10^5^ plaque-forming units (PFUs) per milliliter.

### Plaque assay

Plaque assay was performed on NIH-3T3 or L929 cells ectopically expressing murine CD163, as described previously (18).

### Mice

All mice (Table 1) were obtained from Jackson Laboratories (Bar Harbor, ME, USA) and bred in the Mouse Breeding Core at UW–Madison. Mice of both sexes were inoculated by instilling 50 μL of neat virus stock into the peritoneal cavity, for a dose of 7.1 × 10^8^ N gene copies per mouse and 3 × 10^4^ PFU per mouse. Blood was collected via the cheek-bleed method using a sterile lancet and serum-separator microtainers (BD Lifesciences, Franklin Lakes, NJ, USA).

**TABLE 1:** Mouse strains

Abbreviation	Technical name	Jax ID
WT	C57BL/6J	000664
IFNAR^-/-^	B6(Cg)-Ifnar1^tm1.2Ees^/J	028288
IFNGR^-/-^	B6.129S7-Ifngr1^tm1Agt^/J	003288
IFNAGR^-/-^	B6.Cg-Ifngr1^tm1Agt^Ifnar1^tm1.2Ees^/J	029098
PD1^-/-^	B6.Cg-Pdcd1^tm1.1Shr^/J	028276
RAG^-/-^	B6.Cg-Rag2^tm1.1Cgn^/J	008449
CD45.1	B6.SJL-Ptprc^a^ Pepc^b^/BoyJ	002014

### Viral loads

RNA was extracted from 20 μL of serum using the KingFisher Flex (ThermoFisher, Waltham, MA, USA) with MagMax reagents. Extracted RNA was subjected to RT-qPCR using the TaqMan RNA-to-CT 1-Step Kit (ThermoFisher, Waltham, MA, USA) in a 20 μL reaction with 0.5 μM of primers and 0.1 μM of probe labeled with FAM and ZEN/Iowa Black quenchers (IDT, Coralville, IA, USA). Primer/probe sets for RPgV/maPgV were as follows: forward: TGACTCCGGAGGGATCAATG; reverse: GCATTAATTAGCCGAACAGTGG; probe: TCAGTTTCATGCTTCCAACG. Thermocycling was performed on a Quantstudio 6 Pro (Applied Biosystems, Waltham, MA, USA) with a 96-well block (0.2 mL) under the following conditions: 48°C for 15 min followed by 95°C for 10 min, then 50 cycles of 95°C for 15 s, followed by 60°C for 1 min. An RNA standard was made by cloning a fragment of the LDV genome sequence into the pJET1.2/blunt vector (Invitrogen, Waltham, MA, USA). After linearization of the construct, transcription was performed *in vitro* for 6 hours with the MEGAscript T7 transcription kit (Invitrogen, Waltham, MA, USA), followed by purification using the MEGAclear transcription cleanup kit (Invitrogen, Waltham, MA, USA), quantification, and dilution to a concentration of 1 × 10^10^ transcript copies per microliter. Tenfold dilutions of this transcript were used as a standard curve, which was linear over eight orders of magnitude and sensitive down to 10 copies of RNA transcript per reaction.

## Data Availability

Raw data sets used to make each figure can be found in Dryad, DOI: 10.5061/dryad.2ngf1vj0v.
